# Rupture bilatérale simultanée des deux ligaments croisés du genou: à propos d’un cas

**DOI:** 10.11604/pamj.2017.26.114.11645

**Published:** 2017-03-01

**Authors:** Mounir Yahyaoui, Abdelhafid Derfoufi, Najib Abbassi, Abdelkarim Daoudi, Omar Agoumi, Hicham Yacoubi, Abdeljaouad Najib

**Affiliations:** 1 Service de Traumatologie-Orthopédie, CHU Mohammed VI, Oujda, Maroc

**Keywords:** LCA, LCP, rupture bilatérale simultanée, post-traumatique, Lerat, ACL, PCL, simultaneous bilateral rupture, post-traumatic, Lerat

## Abstract

La rupture bicroisée est rare, des deux LCA simultanément est exceptionnelle, alors que la rupture bicroisée bilatérale simultanée et post-traumatique n’a été jamais décrite dans la littérature, ce qui rend notre cas très intéressant pour étude, suivi et discussion thérapeutique puisqu’on a privilégié la prise en charge thérapeutique en deux temps opératoires espacés dans le temps et nos résultats étaient très satisfaisantes aussi bien pour nous que pour le malade.

## Abstract (en)

Bilateral cruciate ligament rupture is rare while simultaneous rupture in both anterior cruciate ligaments (ACL) is exceptional; that’s why post-traumatic simultaneous bilateral cruciate ligament rupture has never been described in the literature making this case study based on patient’s observation, follow-up and therapeutic discussion very interesting. The procedure was performed in two surgical steps spaced in time and results were very satisfactory for both us and patient.

## Introduction

La rupture simultanée des deux ligaments croisés du genou est une entité assez rare; Cependant leur rupture bilatérale en un seul temps n’est pas du tout décrite ce qui rend notre cas très précieux pour étude et suivi. Nous avons adopté la technique de Lerat modifié pour la reconstruction simultanée des deux ligaments croisés en deux temps opératoires différé de un an.

## Patient et observation

**Figure 1:**
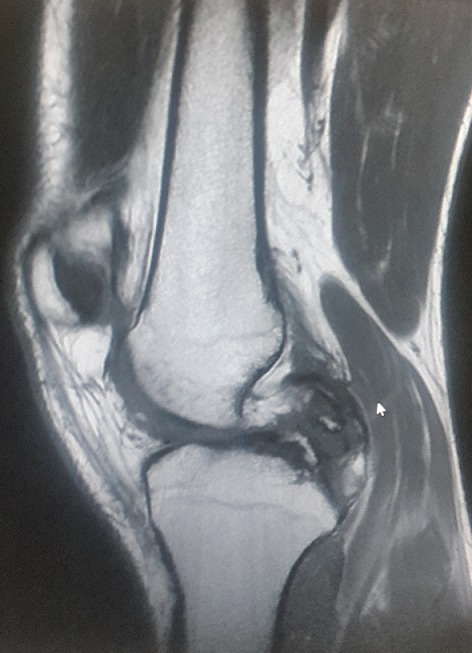
IRM du genou gauche

**Figure 2:**
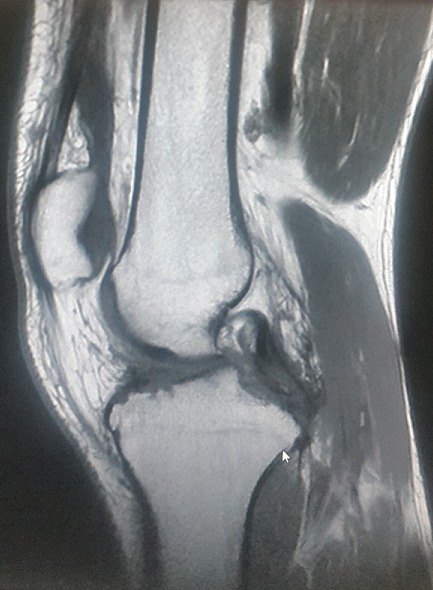
IRM du genou droit

**Figure 3:**
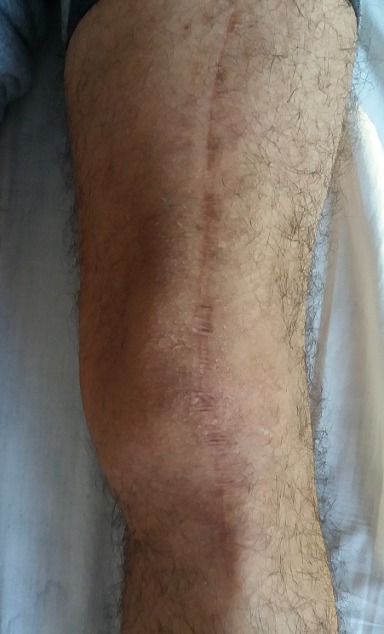
Cicatrice du ligamentoplastie (Lerat) du genou gauche

**Figure 4:**
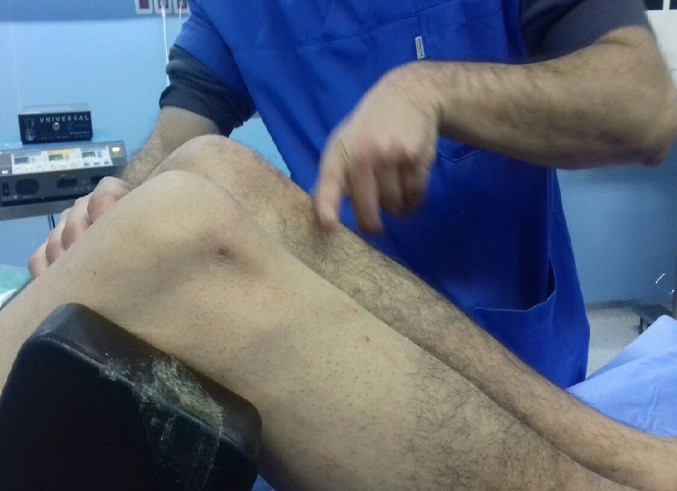
Avalement de la TTA droite

**Figure 5:**
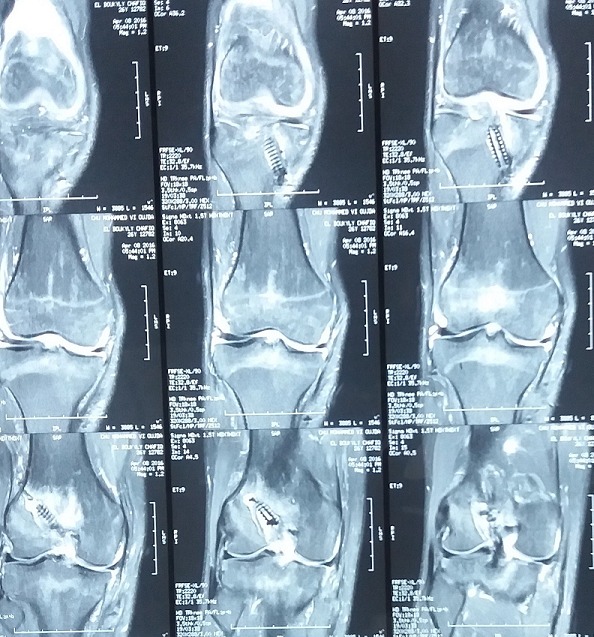
IRM du genou droit post reconstruction du LCA

**Figure 6:**
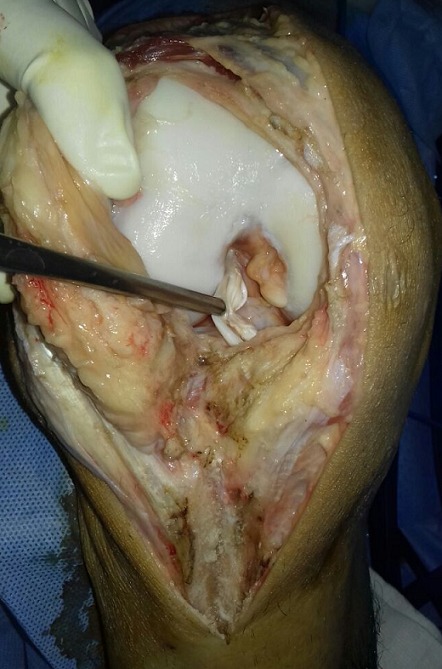
Aspect per-opératoire distendu de notre LCA droit anciennement reconstruit

**Figure 7:**
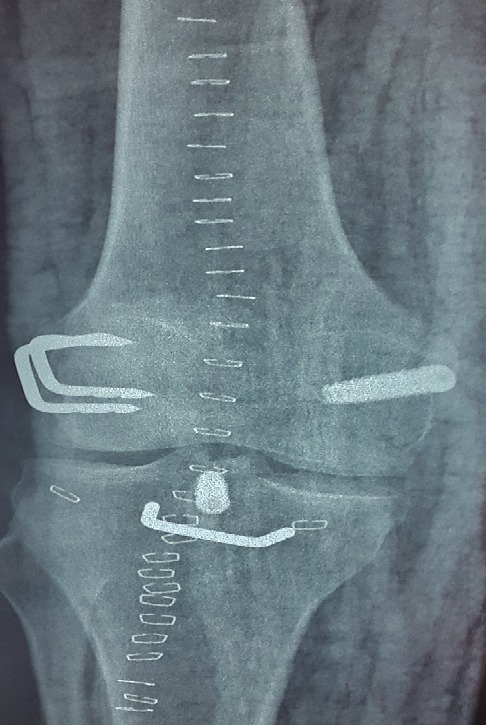
Radiographie post-opératoire du genou droit

Un jeune homme de 25 ans, sportif, sans antécédents, qui a été victime il y a 3 ans d’un accident de la voie publique avec point d’impact au niveau des deux genoux et dont le mécanisme était un tableau de bord. Le patient a consulté aux urgences ou il a bénéficié d’un bilan radiologique standard qui était normal sans lésions osseuses décelables. Un an après, il a consulté dans notre formation pour une instabilité plus marqués au genou gauche. L’examen physique a objectivé une laxité combiné antéro-postérieure du genou gauche sans laxité frontale, pour son genou droit l’examen a objectivé un Trillat Lachman en arrêt mou, alors que le tiroir postérieur n’était pas manifeste. Le bilan radiologique standard était normal, l’IRM du genou gauche (Figure 1) a mis en évidence une rupture bi croisée franche, alors que pour son genou droit, l’IRM (Figure 2) a montré une rupture de LCA, alors que pour le LCP était en hypersignal intra-ligamentaire stade II de Gross. Notre attitude thérapeutique était d’abord de commencer par le genou gauche le plus symptomatique, en réalisant une reconstruction simultanée des deux ligaments croisés par un seul transplant selon la technique de Lerat modifié (Figure 3). Un an par la suite, ce malade était candidat pour un geste chirurgical sur son genou droit ; Après un temps arthroscopique, le LCA était totalement rompu, alors que le LCP était distendu. Notre décision peropératoire était de reconstruire le LCA seul selon la technique de DIDT, et de suivre le malade. Après un an de suivi, notamment après plusieurs séances de rééducation, le malade s’est présenté encore une fois dans un tableau d’instabilité du genou droit (Figure 4), dont l’IRM (Figure 5) a montré un LCA en place mais distendu avec rupture du LCP ; alors on a décidé de réaliser pour ce genou une autre reconstruction selon la technique de Lerat modifié (Figure 6, Figure 7). Nos résultats étaient très satisfaisants pour les deux genoux, et le malade ne présente plus de laxité soit objective ou subjective(score de l’IKDC post-opératoire à 83 pour le genou gauche, et à 79 pour le genou droit).

## Discussion

La rupture simultanée des deux ligaments croisés est une lésion traumatique rare. Elle se voit plus fréquemment dans les traumatismes de haute vélocité en particulier les luxations du genou [1]. Une lésion bilatérale simultanée de LCA est extrêmement rare et n´a été rapportée que trois fois dans la littérature. Une enquête nationale était menéeaux Etas-Unispour déterminer la prévalence des déchirures bilatérales simultanées du LCA et les stratégies de gestion préférées par les chirurgiens orthopédistes de la médecine du sport. Sur 43 réponses, seulement 22 (51,2%) chirurgiens avaient vu une lésion bilatérale simultanée du LCA. Parmi celles-ci, 16 (76,2%) préféraient la reconstitution par étapes. Le choix du greffon a été mélangé entre l´autogreffe et l´allogreffe, mais la majorité a préféré l´autogreffe du tendon rotulien (58%) ou l´autogreffe ischio-jambier (41%). La reconstruction par étapes est le traitement de choix des chirurgiens interrogés dans cette étude [2]. Matjaz Sajovic [3] a décrit le cas d’un patient souffrant de genoux bilatéraux instables à déficience en LCA. La reconstruction bilatérale du LCA était en une seule étape en utilisant les tendons ischio-jambiers. A sept ans de suivi, l’opinion du patient était que les deux genoux reconstitués avaient une fonction normale rejoignant ses activités antérieures. La reconstruction bilatérale du LCA en deux étapes prend beaucoup plus de temps pour le patient et coûte cher pour la santé. Saithna et al. [4] rapporte une série de cas de huit patients ayant subi une reconstruction bilatérale simultanée du LCA, en utilisant des systèmes de pile à deux caméras pour permettre la chirurgie bilatérale réellement simultanée par deux équipes chirurgicales. A deux semaines, tous les patients étaient indépendants dans leur mobilité. Il n´y avait pas de différence entre les scores de changement de pivot, de Lysholm et Tegner à un an par rapport aux résultats publiés pour la reconstruction unilatérale de LCA.

Aucun cas de rupture post-traumatique simultanée bilatérale de tous les ligaments du pivot central n’a été décrit dans la littérature, devant telle situation il faut obligatoirement rechercher une agénésie des deux ligaments croisés. Cette malformation congénitale est exceptionnelle et rarement décrite. Elle se manifeste principalement par une laxité du genou et peut être découverte fortuitement. Le diagnostic peut être déjà fait sur les radiographies standards sur lesquelles il existe toujours une hypoplasie des épines tibiales et une malformation des condyles associée [5] ce qui n’existe pas pour notre cas. L’IRM permet de confirmer ce diagnostic et d’établir un bilan lésionnel afin de rechercher une lésion ou une malformation méniscale. Notre question pertinente était de comment planifier la prise en charge thérapeutique; en un seul temps ou en deux temps opératoires? Du point de vue chirurgical, le traitement des lésions bicroisées demeure toujours controversé. Certains auteurs préconisent l’arthroscopie pour la reconstruction des ligaments croisés [1–6], certains d’autres choisissent la reconstruction du LCP seul [7]. Lipscomb et Anderson avaient rapporté les résultats de 26 patients présentant une rupture des deux ligaments croisés, opérés par arthrotomie. Cette reconstruction pour ces auteurs permet de préserver les structures intra-articulaires notamment les ménisques [8]. Ohkoshi et al. proposent une reconstruction en deux temps, le premier temps consiste en une reconstruction du LCP une fois les conditions locales le permettent, la reconstruction du LCA est faite dans un second temps [9]. Lerat avait décrit sa technique en 1983 [10–11]. Elle consiste en le remplacement du pivot central par un seul transplant et une seule voie d’abord. Une longue bandelette prélevée de l’appareil extenseur comporte à sa partie centrale un fragment rotulien. A partir de ce bloc, les fibres du tendon rotulien remplacent le LCP et les fibres du tendon quadricipital remplacent le LCA. Ce procédé permet aussi de stabiliser le compartiment externe par une plastie extra-articulaire utilisant le même transplant. Le bloc de rotule est impacté en force dans le tunnel tibial. La modification apportée [12] consiste en la préparation d’une gouttière dans le plateau tibial où sera incarcéré le bloc rotulien, ce qui confiera plus de stabilité à l’implant n’étant pas amené à tailler le fragment osseux pour l’adapter au tunnel tibial, ce qui permettra d’éviter de le fragiliser, en plus d’une facilité technique ce qui raccourcira le temps de l’intervention.

## Conclusion

Nous rapportons ce cas extrêmement exceptionnel de rupture simultanée bilatérale des deux ligaments croisés du genou en exposant notre attitude thérapeutique espérant de trouver d’autres cas pour enrichir la bibliographie scientifique de cette précieuse entité.
